# Retention of healthcare workers 1 year after recruitment and deployment in rural settings: an experience post-Ebola in five health districts in Guinea

**DOI:** 10.1186/s12960-021-00596-x

**Published:** 2021-05-17

**Authors:** Delphin Kolie, Remco Van De Pas, Alexandre Delamou, Nafissatou Dioubaté, Foromo Timothée Beavogui, Patrice Bouedouno, Abdoul Habib Beavogui, Abdoulaye Kaba, Willem Van De Put, Wim Van Damme

**Affiliations:** 1 Centre National de Formation et de Recherche en, Santé Rurale de Maferinyah, Forécariah, Ministry of Health, Forécariah, Guinea; 2 Department of Public Health, Institute of Tropical Medicine of Antwerp, Antwerp, Belgium; 3 Department of Health Ethics and Society, Faculty of Health Medicine and Life Sciences, Maastricht University, Maastricht, The Netherlands; 4 Department of Public Health, University of Conakry, Conakry, Guinea; 5 Bureau de Stratégie Et de Développement, Ministry of Health, Conakry, Guinea

**Keywords:** Retention, Absenteeism, Healthcare workers, Post-Ebola, Rural Guinea

## Abstract

**Background:**

Guinea undertook health workforce reform in 2016 following the Ebola outbreak to overcome decades-long shortages and maldistribution of healthcare workers (HCWs). Specifically, over 5000 HCWs were recruited and deployed to rural health districts and with a signed 5-year commitment for rural medical practice. Governance structures were also established to improve the supervision of these HCWs. This study assessed the effects of this programme on local health systems and its influence on HCWs turnover in rural Guinea.

**Methods:**

An exploratory study design using a mixed-method approach was conducted in five rural health districts. Data were collected through semi-structured questionnaires, in-depth interview guides, and documentary reviews.

**Results:**

Of the 611 HCWs officially deployed to the selected districts, 600 (98%) took up duties. Female HCWs (64%), assistant nurses (39%), nurses (26%), and medical doctors (20%) represented the majority. Findings showed that 69% of HCWs were posted in health centres and the remaining in district hospitals and the health office (directorate); the majority of which were medical doctors, nurses, and midwives. The deployment has reportedly enhanced quality and timely data reporting. However, challenges were faced by local health authorities in the posting of HCWs including the unfamiliarity of some with primary healthcare delivery, collaboration conflicts between HCWs, and high feminization of the recruitment. One year after their deployment, 31% of the HCWs were absent from their posts. This included 59% nurses, 29% medical doctors, and 11% midwives. The main reasons for absenteeism were unknown (51%), continuing training (12%), illness (10%), and maternity leave (9%). Findings showed a confusion of roles and responsibilities between national and local actors in the management of HCWs, which was accentuated by a lack of policy documents.

**Conclusion:**

The post-Ebola healthcare workers policy appears to have been successfully positive in the redistribution of HCWs, quality improvement of staffing levels in peripheral healthcare facilities, and enhancement of district health office capacities. However, greater attention should be given to the development of policy guidance documents with the full participation of all actors and a clear distinction of their roles and responsibilities for improved implementation and efficacy of this programme.

**Supplementary information:**

**Supplementary information** accompanies this paper at 10.1186/s12960-021-00596-x.

## Background

The situation of skilled healthcare workers (HCWs) in Guinea is characterized, on one hand, by a critical shortage, and on the other hand, a disparity in their distribution between rural and urban settings [1]. For instance, in 2013, before the Ebola outbreak, the density of skilled HCWs in Guinea was estimated at 7.3 per 10,000 inhabitants; meaning that nearly only 8000 skilled HCWs were available to cover the 11 million population. This ratio was three times lower compared with the threshold of 23 HCWs per 10,000 inhabitants required by the World Health Organization (WHO) in 2006 [1, 2]. In addition, only 30% of these HCWs stayed in rural areas where 70% of the Guinean population live [4]. Furthermore, 55% of these personnel worked in the capital Conakry where only 15% of the population live [3]. In previous studies, we had tried to understand the contextual factors underlying the shortage of HCWs in Guinea and their low presence in rural areas [4–6]. Results revealed five underlying and interconnected factors. The first factor is the low employment capacity of the state, contrasting with the chronic over-supply of health graduates on the labour market—more than 25,000 skilled HCWs trained between 2010 and 2017, while nearly 12,000 job vacancies existed [3, 4, 7]. Consequently, many of these health graduates stay in the capital, Conakry where access to the formal or informal private sector is easier. A relatively low number of them, however, stay in rural health districts, where they have been working for years as informal HCWs (e.g. volunteer or contractual workers) in public health facilities with the prospect of being prioritized by state actors in recruitment processes [4]. These HCWs play a pivotal role in healthcare delivery in rural and underserved health districts, where they make up 68–71% of the overall (formal and informal) workforce [4]. The second factor is related to the under-notification of HCWs in the country. In fact, despite the role informal HCWs play in the health system, only public HCWs^1^ are reported in official payrolls of the workforce [4, 6]. The third factor is linked to the centralized-type of HCWs governance resulting in their inequitable recruitment and management, and high turnover of public HCWs who are often recruited from the capital, Conakry [4, 5]. In addition, the lack of financial and non-financial incentives for rural practice, the discriminatory management (access to continuous training and administrative position), and the high workload of HCWs staying in rural areas do not favour their retention in these settings [6]. Finally, factors acting outside the health system such as inadequate living conditions in rural settings negatively influence the presence of HCWs [6].

Nonetheless, in the aftermath of the Ebola outbreak, efforts were made by the Guinean government, with the support of development cooperation initiatives, to strengthen the health system, and to make it resilient to future epidemics, through improved availability and distribution of HCWs [5, 8]. Initiatives that resulted from this reform allowed the recruitment of over 5000 HCWs in 2016 and 2017 and their majority deployment (95%) to rural health districts in April 2017 [5]. In addition, and as recommended by the WHO, exceptional regulatory and financial measures were applied to this new staff in order to increase their motivation for working in their assigned areas and, therefore, limit their turnover [5]. These measures include the signing of a five-year contract for rural practice; a 40% increase in their salary; the delocalization, at the district level, of the salary payment; and the creation of a directorate of human resources for health within the Ministry of Health (MoH) with more responsibilities in this staff supervision [4, 5]. Furthermore, international actors have established a budgetary-support to the post-Ebola HCWs reform, but conditional to the achievement of a minimum retention rate of 80% of HCWs in their assigned locations [5]. Some other initiatives were undertaken in the selection, deployment, and management processes of these new public HCWs in the post-Ebola context as shown in Box 1.

### Box 1: Public HCWs recruitment and deployment processes in Guinea

**Table:** 

Key processes	Pre-Ebola context	Post-Ebola context
Recruitment	HCWs selected through a national test organised by the Ministry of Public Services (MoPS)	By August 2016, nearly 3,000 HCWs recruited nationwide through a test organized by the MoPS By December 2016, nearly 1,000 HCWs recruited by the MoPS in conjunction with the MoH for their participation in the Ebola response and control programmes In 2017, more than 1,000 HCWs recruited by the MoPS in conjunction with the MoH from health facilities (unknown selection criteria) Condition for services of HCWs recruitment undertaken: HCWs signed a five-year commitment for medical practices in rural areas
Deployment	Deployment of public HCWs in health districts and their posting in health facilities ensured by the MoH	Deployment in health districts and posting to health facilities done in two stages: The MoH deployed public HCWs in health districts Local health district authorities ensured posting at the health-facility level
Management	Salary payment conditioned to the submission of proof (or acts) of duty take-up to the Ministry of Finance (MoF) by the public HCWs Duty take-up delivered by local health districts authorities HCWs wages directly wired to their bank account by the MoF	Salary payment conditioned to the submission of proof (or acts) of duty take-up to the MoF by the public HCWs Duty take-up delivered by local health districts authorities HCWs wages transferred to local health districts authorities who proceed to payment (physical presence required)

Therefore, the main objective of this study was to document the implementation process of this post-Ebola HCW retention policy at the local (district) level and assess to what extent this policy would influence the turnover of HCWs in rural Guinea. Three main research objectives were identified:

1. To analyse the effects of the post-Ebola deployment of public healthcare workers on local health systems and services;
2. To analyse barriers and enablers of the retention of public healthcare workers in rural areas;
3. To assess the turnover of public healthcare workers one year after their deployment.

The relevance of this research resides in its potential for providing information on factors influencing the retention of HCWs in the post-Ebola context in rural Guinea. Such findings would support policy-making and programming and guide the post-Ebola Health Systems Strengthening (HSS) reform.

## Methods

### Analytical framework

**Fig. 1:**
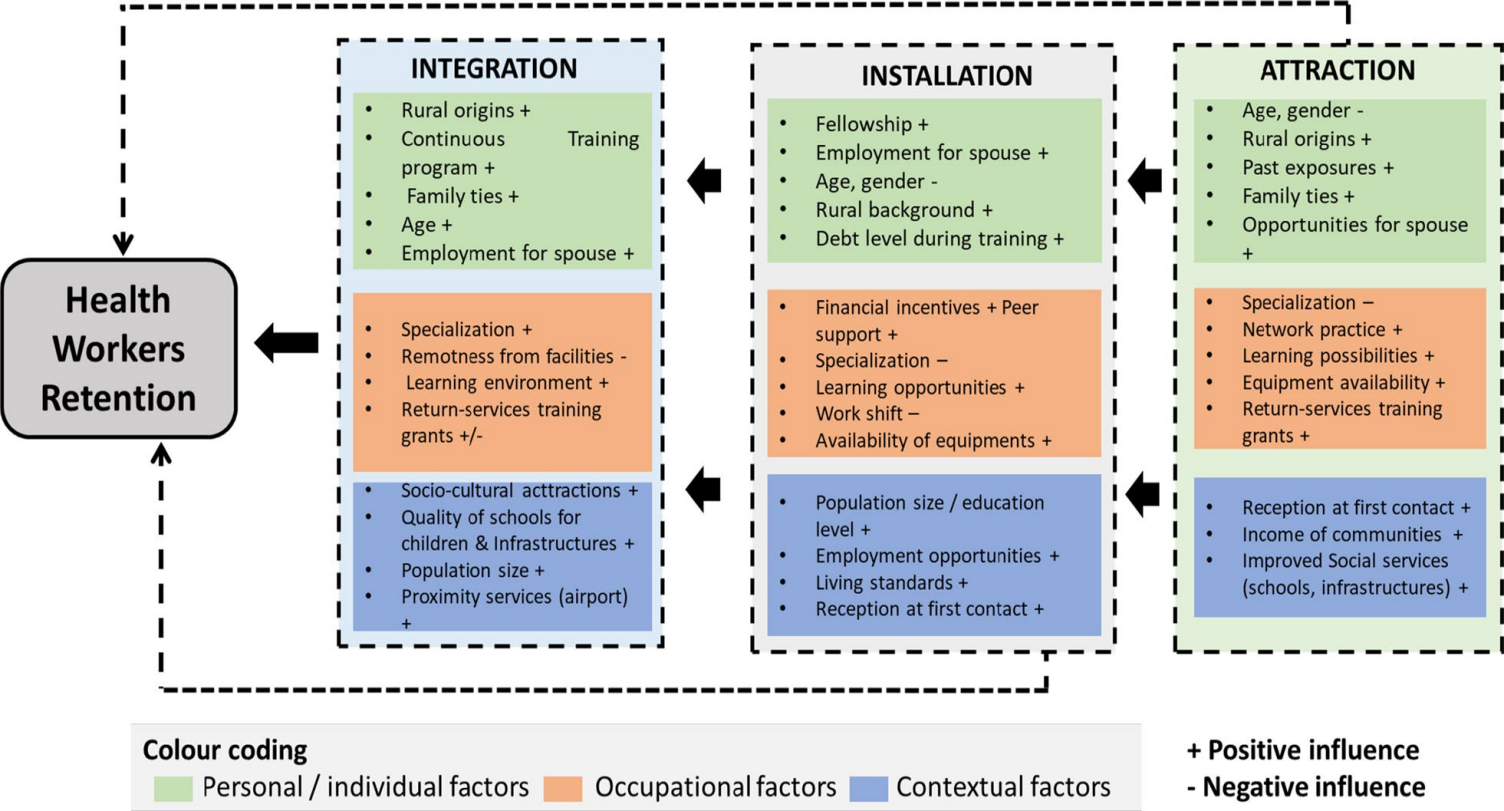
Adapted from Bilodeau’s analytical framework of the main factors determining health workers’ attraction, installation, and integration in remote areas

The Bilodeau analytical framework (as shown in Fig. 1) was adapted to guide the analysis of barriers and enablers of the retention of HCWs in rural Guinea in the post-Ebola context (research objective 2). This framework was used as it provides information on how the decision of a HCW to settle in a given area could be affected over time.

The model describes the retention of HCWs in rural and remote areas as the result of a three-stage decisional phase: attraction, installation, and integration [9].

- Attraction refers to “a positive attitude regarding the exercise of medicine in rural areas which does not necessarily lead to installation”;
- Installation consists of the realization of attraction and the decision to settle and practice in a given area;
- Integration is defined as a result of experiencing living and working conditions in a determined area.

At each decisional phase, various personal or individual, occupational or professional, and contextual or environmental factors influence the experience of HCWs and consequently, their decision to leave, or not, a given location [9].

### Study setting

**Fig. 2:**
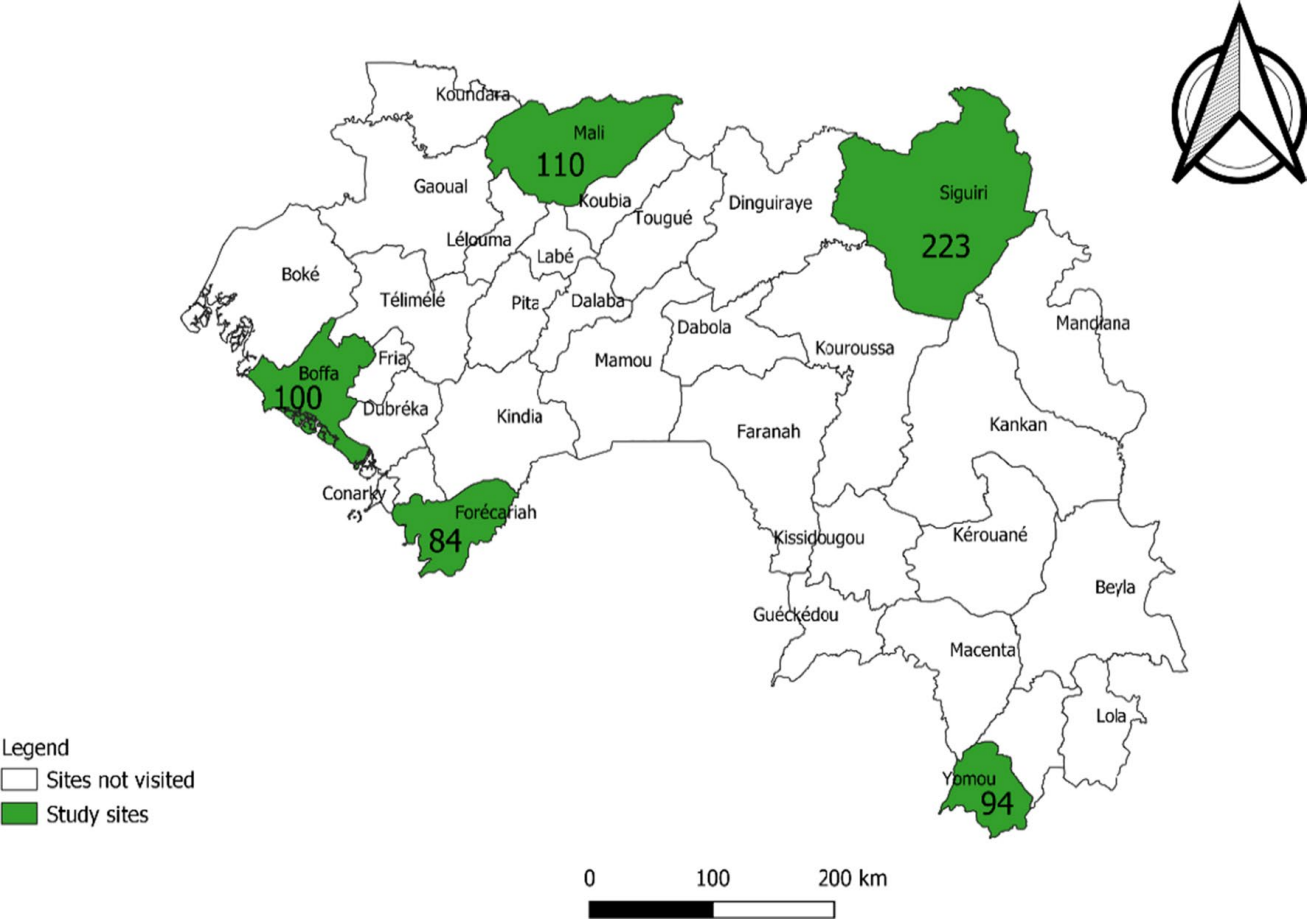
Location of study sites and the number of healthcare workers officially deployed in April 2017

We selected the regions of Boké, Kindia, Labé, Kankan, and N’zérékoré because of their critical shortages of HCWs compared with other regions of the country. In each of these five regions, we purposively selected one health district based on differences in health indicators, geographical accessibility, demographic characteristics, and local living conditions (Table 1). Explanatorily, some of the study sites are easily accessible from the capital, Conakry (Boffa and Forécariah), economically attractive (Siguiri), while others are relatively isolated (Mali and Yomou) [10, 11]. Therefore, the selection of health districts with different characteristics would help to generate variability in factors affecting the turnover of HCWs in rural Guinea in the post-Ebola context. Overall, 611 HCWs were deployed, by the Ministry of Health (MoH), to the five health districts by April 2017 (Fig. 2).

**Table 1:** Background information on study sites socio-economic, demographic, geographic and health system characteristics, Guinea, May 2020 [10]

Characteristics	Forécariah	Boffa	Mali	Siguiri	Yomou
Region of:	Kindia	Boké	Labé	Kankan	N’zérékoré
Regional population size	1 813 979	1 259 075	1 154 296	2 281 221	1 834 758
Number of HCWs available in 2013 at regional level	1030	660	779	645	841
Sources of income	Illegal diamond mining, trade, agriculture, animal breeding and fishery	Bauxite mining, Agriculture, animal breeding and fishery	Agriculture and animal breeding	Gold mining, agriculture, animal breeding and fishery	Agriculture, animal breeding and fishery
Population under the poverty line	62%	59%	65%	49%	67%
Number of districts administered	5	5	5	5	6
Adult literacy	30%	32%	23%	18%	27%
Health indicators^**a^					
Immunisation coverage of under 1 year children	12%	17%	8%	36%	35%
Health service utilisation	18	13%	15%	12%	18%
Anaemia in children 6–59 months	75%	69%	71%	78%	76%
Assisted birth delivery	43%	39%	44%	61%	72%
Geo-demographic characteristics					
Distance from the capital, Conakry (Km)	100	146	557	771	1,029
Population density (*inhabitants per square km)*	62	47	36	61	32
Population in rural/remotes zones	91%	96%	98%	80%	93%
Level of isolation	Good	Very good	Very poor	Good	Very poor
Health facilities	1 hospital, 10 health centres, 4 formal private facilities	1 hospital, 8 health centres, no formal private facility	1 hospital, 13 health centres, 2 formal private facilities	1 hospital, 15 health centres, 10 formal private facilities	1 hospital, 7 health centres, 3 formal private facilities

^a^Regional health indicators used as a proxy for district health indicators

### Study design

This was an exploratory study design using a mixed-method (quantitative and qualitative) approach [12]. Because of the research question, priority was given to the qualitative component of the study.

### Sampling and recruitment of participants

A stratified purposive sampling technique was used to guide the selection of study participants for the qualitative component of this study [13]. The first stage of this technique consisted of the identification of five sub-groups of study participants: (1) local government officials, (2) local health authorities, (3) community representatives, (4) local health facilities and services managers, and (5) healthcare workers (former and new). Participants were then selected from each of this sub-group in a purposive manner to ensure that the sample was of maximum variation, that is, participants were selected as much as possible, according to the heterogeneity of their age, gender, education level, years of experience, and professional practices. [14] This sampling technique allowed us to describe and analyse in detail perceptions within and across the sub-groups.

For the quantitative component, HCWs under the post-Ebola retention programme that were present at their post during data collection were systematically enrolled in this study.

### Data collection

Data were collected using a questionnaire, an in-depth interview guide, and a documentary analysis from April to June 2018.

Two researchers from the Maferinyah National Research and Training Centre (CNFRSR), and respectively 10 research focal points and medical graduates (two per health district) conducted 120 in-depth interviews with local government officials (4%), local health authorities (17%), community representatives (10%), health facilities and services managers (27%), and healthcare providers (42%). All interviews were conducted in French. Research focal points and medical graduates stayed in the study sites for two months, from 25th April to 29th June 2018, and collected data on absenteeism in two main stages. The first stage consisted of the interview of all new HCWs present at their post at the first passage of the data collectors. The second stage consisted of the inclusion of any new HCWs absent at the first passage but who showed-up within the two months of the data collection.

The research focal points included HCWs under the post-Ebola retention programme with great interest in the study, and holding positions of responsibility: responsible for health human resources, planning, research, and training units at the district level. The medical graduates were selected from the Public Health Department of the University of Conakry and part of the study data were used for their theses. All the research focal points and medical graduates were trained on the research protocol and the data collection tools. The pre-test of data collection tools was conducted in health facilities in locations different from study sites.

The documentary review focused on official acts, texts, payroll records, and HCWs’ registries available at the study sites.

### Data analysis

Interviews were transcribed and coded manually following a thematic analysis approach [15]. Data were thematically organised and analysed based on the research objectives: (1) perceptions and experiences about the effects of deployment, (2) factors influencing the attraction, installation, and integration of HCWs, (3) underlying factors of the turnover of HCWs, and (4) proposed solutions for improving the on-going retention policy (details of the interview guide is found in Additional file 1: Annex S1).

Data from the questionnaire were entered into a dedicated EpiData database (version 3.1 for entry EpiData Association, Odense, Denmark) and summarised using proportions or means (± standard deviation).

### Key variables definition

A skilled HCW is a health professional holding academic and practical training from a public or private institution accredited by national, regional, or international standards. HCWs considered skilled in Guinea include medical doctors, nurses, midwives, health technicians, etc. HCWs turnover was understood as a result of job dissatisfaction, which can be measured by HCWs’ absenteeism, intention to quit, and the demotivation to stay [16]. As for absenteeism, it was defined—referring to the public expenditure tracking survey—as the unavailability of new HCWs at their assigned health districts during the data collection period (two months) [17–19]. This method consisted of asking HCWs found on duty to enumerate all absent colleagues and to provide reasons as to why they were absent. Knowing that this method is prone to social desirability and reporting biases, and to facilitate identification of new HCWs, the research team was provided with a list of all new HCWs per district and health facility [20]. We assumed that, no matter the reasons for absenteeism, if a HCW fails to attend his/her post for two months, his/her likelihood of being outside (mobility/turnover) his/her assigned health district is high. This definition took into account the fact that these new HCWs undergo special payment and management measures (Box 1). The term “medicalization of health centre” was used to define the posting of a medical doctor at a primary health facility.

## Results

Based on the study objectives that guided the data analysis, the results are presented below according to the different emerging themes.

### Effects of the deployment of HCWs on local health systems

Out of 611 HCWs officially deployed in the selected rural health districts in April 2017, 600 (98%) took-up duties. The distribution characteristics of these HCWs are depicted in Table 2.

**Table 2:** Distribution of health workers who took-up duties in the five selected rural health districts, *N* = 600

Variables	Medical doctors	Nurses	Midwives	Assistant-nurses	Other cadres^a^	Total Number (%)
Levels of health facilities						
District health office	25	2	**–**	**–**	1	28 (5)
District hospital	44	43	17	39	14	157 (26)
Health centres^b^	49	111	53	193	9	415 (69)
Gender						
Female	43	88	70	173	12	386 (64)
Male	75	68	**–**	59	12	214 (36)

^**a**^Pharmacists, dentists, biomedical, Lab technicians, public health technicians

^**b**^In total, there exist 53 functional health centres in the selected districts

Female HCWs accounted for 64% of them. Assistant-nurses (39%), nurses (26%), medical doctors (20%), and midwives (12%) were the most represented socio-professional categories.

Overall, HCWs were mostly posted in health centres (69%) and district hospitals (26%). Moreover, 42% of medical doctors (49 out of 118), 71% of nurses, and 76% of midwives were assigned to health centres.

Two main themes emerged from the interviews with participants when asked about the potential effects of the deployment of HCWs.

First, respondents stated that the Ebola Virus Disease outbreak led to job-abandonment of many unemployed (volunteer) HCWs because of the fear of contracting the disease or the take-up of employment offers in epidemic control programmes, especially with international organizations. As such, according to them, the deployment of new HCWs helped to fill this gap and improved staffing levels. This finding correlated with quantitative data on publicly funded HCWs available on the study sites before and after the deployment of new HCWs in 2017, wherein as much as a 60% increase in staffing levels was reported (Table 3).

**Table 3:** Distribution of publicly funded Healthcare workers per health district in 2017, before and after the deployment

Year	Publicly funded Healthcare workers deployed per health district					
	Boffa	Forecariah	Mali	Siguiri	Yomou	Total
2016	72	66	38	189	44	409
2017	172	150	148	412	138	1020
Increase in %	58	56	74	54	68	60

Second, according to participants, the deployment allowed the staffing of managerial positions with qualified HCWs owning several competencies, for example in computer processing, teaching, and care delivery. This, according to participants, resulted in better care organization through the sharing of tasks and workload among the staff but also in timely reporting of completed and good quality data.

*... We have fully staffed, for the first time, our organic framework… Today many technical problems with our computers, or the making of patients’ consultations notebooks are solved on the spot by this personnel... Now, the units of statistics, planning, and training are held by medical doctors across the country... It helps to the rapid [timely] reporting of completed and good quality data... (IDI# 28 local health authority)*

However, five challenges were reportedly faced by local health authorities, health facilities, and services managers during the posting process of HCWs.

First, participants reported that many HCWs were unfamiliar with the delivery of primary healthcare in rural settings. This, according to them, was due to the fact that few had experience and acquaintance with medical practice or were rather accustomed to the delivery of care in hospitals and urban settings. Some local health managers reportedly organized intensive on-site training for HCWs, on the functioning of primary healthcare facilities, especially immunization and maternal and primary curative care.

Second, the high proportion of female HCWs was another reported challenge as many were pregnant or breastfeeding at the time of the deployment. This pushed local health authorities to predominantly (re-) assign female HCWs to urban or easy-to-access rural health facilities and to compensate for it, unemployed HCWs (mainly males) were reallocated to rural and hard-to-reach areas with some incentive packages (e.g. allocation of 10–20% of healthcare facilities monthly income).

Third, some local unemployed HCWs left public health facilities as a result of their frustration for not been recruited.

*... We have worked for several years here but the district health director never manages to recruit us as public servants... We [eight of us] decided to create our private cabinet... We see over 300 patients per month [more than most health centres in the locality do] and the income we generate daily [from patients] is used to buy food and eat together... Patients prefer coming to us because of the trust we built with them over the years we were working in the public health facilities... (IDI# 11 health provider)*

Fourth, participants reported collaboration conflicts between new and former HCWs. These were mainly sustained, according to participants, by the perceived unequal treatment of HCWs vis-à-vis appointment at positions of responsibility which were in favour of new HCWs. This impacted healthcare organization and functioning in some health districts, especially in the conduction of health services monitoring and evaluation activities.

To address collaboration conflicts, some local health authorities carried out a complete reshuffle of management positions in the frontline health facilities including sending former HCWs, especially health services managers, to places unfamiliar to them.

Finally, it emerged from interviews that HCWs were posted in local health facilities without a proper task description. According to respondents, this resulted in less accountability of HCWs and reportedly accentuated collaboration conflicts between them, as quoted below.

*... It is the traditional birth attendant and assistant-nurses who have being delivering births here and that has not changed even with our presence [after our assigning]... But it is difficult to do anything in this situation because no written document distinguishes the roles of an assistant-nurse, a nurse, or a midwife... We have been parachuted here without any documentation on our roles and responsibilities and this limits us in our work and the claiming of our rights... (IDI# 6 health provider)*

### Barriers and enablers of the retention of health workers

This section on the barriers and enablers of HCWs retention follows the elements of attraction, installation, and integration, according to the Bilodeau analytical framework.

### Attraction factors

**Fig. 3:**
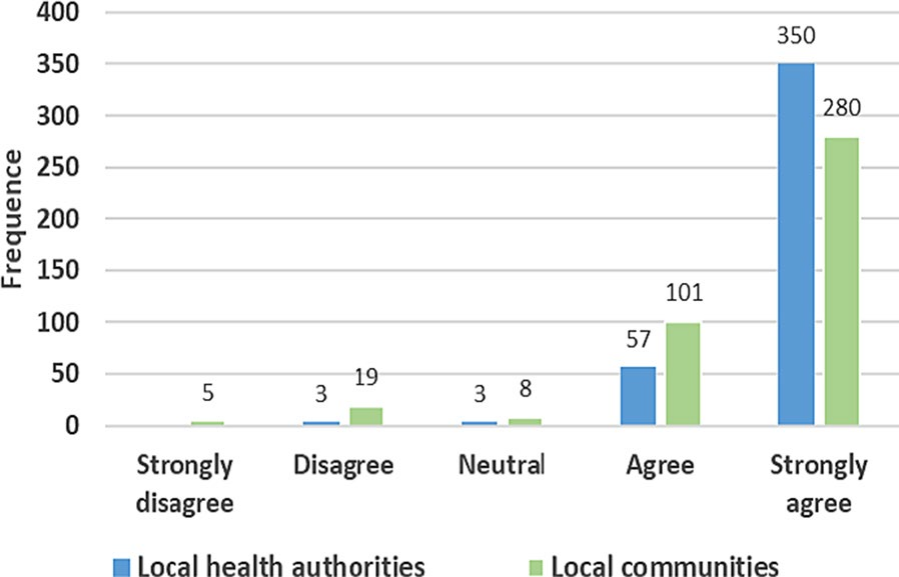
Frequency distribution of responses to the question: ‘’ When you came to take-up office, were you well received by the local health authorities’’ and ‘’ local communities’’? *N* = 413

Overall, 85% of the new HCWs reported that they were well received by local health authorities. Totally, 68% of participants were also reportedly well received by the local communities during the take-up of duties (Fig. 3).

From the interviews, three main reasons emerged as factors attracting HCWs in rural Guinea in the post-Ebola context.

First, the desire to become a public servant was an important attraction factor for rural practice. For some participants, the advantage related to the status of a public servant is that it provides a lifetime guarantee and requires fewer constraints and working time as usually required by the private sector. For some other HCWs, it offers an opportunity to become independent from the family or spouse after several years of dependency and its related-financial implications in, for instance, supporting education and living expenditures.

*… Public servant status gives a lifetime guarantee… You can still be paid even when you are sick or retired… Working in the private sector represents a risk even though it pays well… (IDI#12 health provider)*

Second, working in rural areas was perceived by HCWs as an opportunity to continue practising medicine and learning from another context. For some HCWs interviewed, especially nurses and midwives, health facilities in rural areas are less staffed compared with urban settings, and in that sense, offer unique learning opportunities. For instance, some nurses and midwives reported that working in rural areas provides more responsibility and helps to acquire knowledge that is only dedicated to medical doctors in urban places.

*... As a nurse, practising in rural areas gives a lot of learning opportunities... Here I have been taught how to examine a patient including using a stethoscope but in the national hospital I worked before, I could only inject patients or ensure their nursing... (IDI#38 health provider)*

Third, HCWs stated that they were attracted by rural practice because of their vocation to help disadvantaged populations like those living in rural areas. They justified this by their involvement in humanitarian projects in rural areas, be it their actual assigned zone or not. This group of HCWs is interested in working anywhere if the need arises.

### Installation factors

The role of local health authorities and community representatives were repeatedly mentioned by HCWs as a favouring factor for their installation, particularly in rural areas. In some places, the facilitation of accommodation acquisition, the donation of food and cooking utensils to HCWs, and provision of means of transport (administrative and personal) was reported.

However, difficulties were reported by some HCWs during their installation. These were related to lack of housing especially in urban and mining areas, the working atmosphere, and local living conditions (roads, food, etc.).

First, participants reported difficulty in obtaining houses.

It was reported that HCWs stayed in health facilities for a while before getting accommodation. Because of this, some HCWs temporarily left their assigned post. These participants highlighted that they were afraid of contracting nosocomial infections while they also had financial difficulties in bearing the costs of housing. They suggested that the state give installation bonuses to facilitate the HCWs installation process in rural areas.

Second, some HCWs reported collaboration conflicts with the former workforce in their assigned positions. This, according to them, existed because the former staff considered them a threat to their position of responsibility. The role of HCWs’ parents was crucial, at this stage, for motivating them not to leave their posts.

*... It was not easy to work with them... It was not easy at all... I went back to Conakry and informed my parents ... They are the ones who encouraged me to come back to my post... My mother told me, it is a public service, not someone's property, you cannot leave your job because of someone’s attitude towards you... (IDI#34 health provider)*

Other attitudes were also adopted by HCWs to cope with this situation; the most important of which were the exclusive focus on the provision of care (financial management left to the former staff) and the avoidance of calling oneself a civil servant.

Third, with regards to living conditions, we quote a HCW below:

*... I was assigned to “Mixi” [fictitious name]... Cars only go there once a week... When I arrived in the village [place of deployment], I cried and I said to myself—why did the state do this to me?... My first 2 nights here, I didn't sleep at all... I took it as a punishment from the state... (IDI#12 health provider)*

### Integration factors

**Fig. 4:**
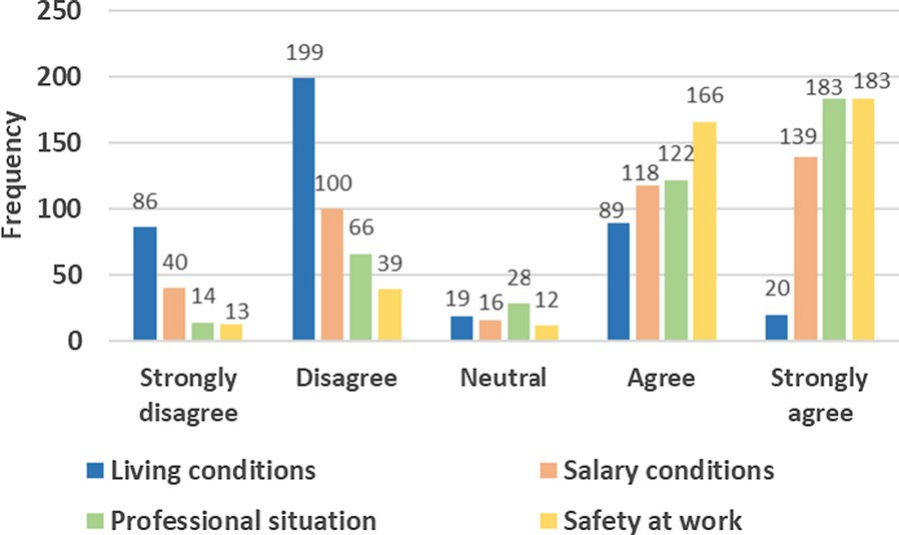
Frequency distribution of responses to the question: “ Are you satisfied with your current living conditions (water, electricity, food, road, internet)”, Salary conditions “professional situation, and is there an environment of safety at work”? *N* = 413

Up to 69% of HCWs surveyed were not satisfied with their living conditions. However, respectively, 62% and 74% were satisfied with their salary conditions and professional situation, and 85% felt secure at work (Fig. 4).

Poor living conditions, low salaries, and limited learning environments were mainly identified as factors impeding the integration of HCWs in rural areas. With regards to living conditions, the difficulties of accessing schools for children, electricity, potable water, internet, and decent housing were considered major obstacles.

*… Here is an island, there is no potable water… The water of the wells is salty; we have to buy packets of water in town [sub-district located 15 km away]… (IDI#17 health provider)*

Issues such as difficulties for children to readapt to the new situation of the family, the fragile health status of a family member, and inaccessibility of the currently assigned zone were also reported. From some married female HCWs’ point of view, staying far away from ones’ husband and family is not well perceived in the Guinean context.

*... Being married and staying away from your husband for months is not well perceived by society... People often downgrade you... They think you are in extramarital relationships with your colleagues or managers or that you have no sense of social value... If you have a jealous husband or an annoying family-in-law, they will always ask you to choose between your work and your family... (IDI#42 health provider)*

Salaries were a major concern for many participants but with a different effect depending on individual characteristics. For HCWs with many people to care of (including their children) and previous exposure to private practice (including working with international organizations before their recruitment), the major concern was the insufficiency of salaries along with the lack of alternative sources of income in rural areas to cover actual expectations and needs of the family.

In some study sites, local health facilities managers were allocating financial bonuses to HCWs to compensate for low salaries. This included the sharing of 10–20% of the healthcare facilities’ monthly income to HCWs as motivational bonuses. Exceptionally, in some mining zones, health facilities managers authorised HCWs to sell their medicines during night-shifts, but under the supervision of a regulatory committee that controls drug quality and sale prices.

For HCWs posted in rural and hard-to-reach areas, the lack of financial compensation for geographic distances and difficult living and working conditions were reported as factors inhibiting their integration. For example, some HCWs complained that the current salary payment method was creating inequality concerning the disadvantages of HCWs posted in hard-to-reach areas compared to their peers working at the district level. HCWs posted in hard-to-reach settings have to pay part of their salaries as transportation fees for accessing them at the district health office—salaries are paid, by cash, at the district health office—while their colleagues staying in urban areas are not exposed to such extra expenses.

Other elements inhibiting the integration of HCWs were the under-utilisation of health services by communities in rural areas. According to participants, the population in remote areas utilise health services once a week and exceptionally during three months of the rainy season—which corresponds to high malaria transmission period. Many midwives also supported that birth deliveries are attended in communities, by traditional birth attendants, and only a few of them are referred to them at the stage of complications.

### Turnover of healthcare workers

#### Absenteeism

**Fig. 5:**
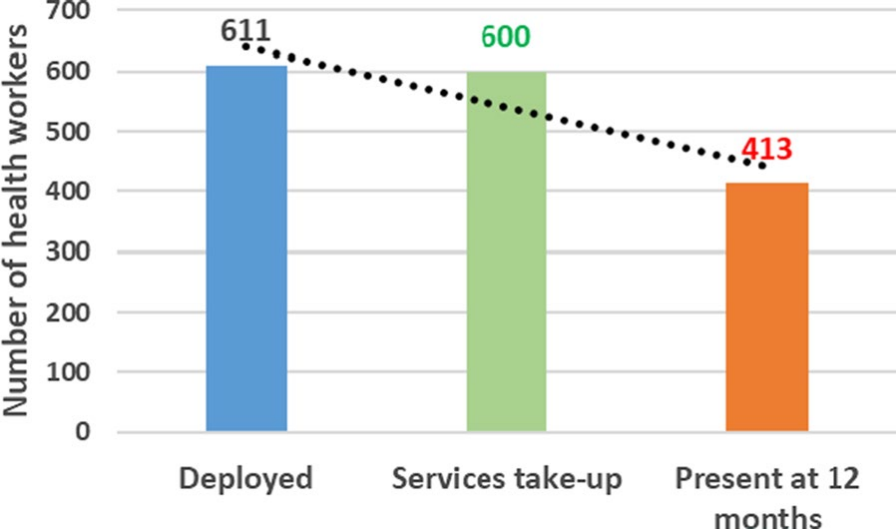
Frequency distribution of health workers present at their assigned post one year after deployment

A total of 413 (69%) HCWs were present at their assigned posts 12 months later. This represented a 31% absenteeism rate among recently deployed HCWs (Fig. 5).

The sociodemographic characteristics of the HCWs present at their posts 12 months after their deployment are presented in Table 4. Absenteeism rates were more pronounced among female HCWs (33%), medical doctors (47%), nurses (31%), and midwives (29%).

**Table 4:** Profile of health workers present at their assigned posts 12 months after deployment, (*N* = 413)

Variables	Before 1 year number (%)	After 1 year number (%)
Age (years)		
≥ 39		378 (91)
< 40		38 (9)
Mean [SD]		33 (4)
Sex		
Female	386 (64)	259 (63)
Male	214 (36)	154 (37)
Marital status		
Married/In couple		359 (87)
Single		46 (11)
Widowed		5 (1)
Divorced		3 (1)
Socio-professional cadres		
Assistant-nurses	232 (39)	169 (41)
nurses	156 (26)	108 (26)
Medical doctors	118 (20)	63 (15)
Midwives	70 (12)	50 (12)
Other cadres^a^	24 (4)	23 (6)
Usual residence corresponds to assignment district		
Yes		139 (34)
No		279 (66)
Actual assignment district meets expectations		
Yes		213 (52)
No		200 (48)
Preference district of assignment, *n* = 200		
Another district within the region of current assignment		116 (58)
Conakry		70 (35)
Another district outside the region of current assignment		14 (7)
Recent history of redeployment		
Yes		12 (3)
No		401 (97)
Occupation of the spouse, * n* = 359		
Civil servant / employed		169 (47)
unemployed		73 (21)
Workmen		66 (18)
Trader/seller		51 (14)
Stay with the spouse, * n* = 359		
Yes		157 (44)
No		202 (56)
Number of people in charge		
≤ 5		141 (34)
> 5		242 (66)
Mean [SD]		7 (4)

^a^Dentists (2), pharmacists (2), biomedical (9), lab technicians (8), public health technicians (2)

### Reported reasons for absenteeism

**Fig. 6:**
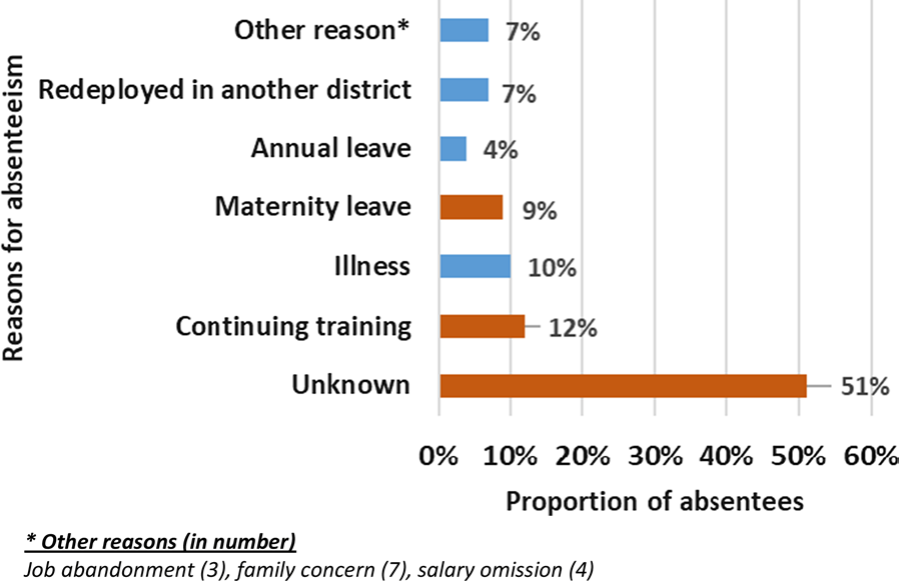
Reasons for absenteeism one year after deployment in five rural health districts in Guinea, *N* = 187

Figure 6 depicts the reasons for absenteeism of new HCWs. The reason for absenteeism from work was unknown/non-justifiable in 51% of cases meaning that these HCWs left their posts without prior request and authorization of their supervisors. Continuing training (12%), illness (10%), maternity leave (9%), and redeployment to another health district (7%) were the other most common reasons for absenteeism.

None of the above reasons were documented at local levels, and interviews revealed an underlying factor: the lack of transparency and fairness in the decision-making of health system actors both at the central and local levels.

First, actors from the central and regional levels of the MoH were reportedly influencing decision-making processes regarding the management of some HCWs. For instance, participants reported that training leaves and redeployment acts were exclusively delivered by the central administration of the MoH and without prior consultation of local health authorities. Also, according to participants, some decision-makers from the central and regional levels were involved in leave-requests concerning their relatives, especially in the district surrounding the capital Conakry.

*... More than 80% of the health personnel affected in Coyah, Boffa, Kindia, and Forecariah were women... And most of them have their husbands working in different ministries and departments in Conakry... What can a health centre or a district health manager do when, for instance, a national director asks him to allow his wife to join him in Conakry for whatever reason? (IDI# 8 local health manager)*

Second, at the district level, maternity and annual leaves were allocated by local health authorities without proper coordination with community representatives, the local administrative authorities, and sometimes, local health facilities and services managers. Because of this, according to participants, some HCWs were exceptionally paid through money transfer platforms and thus, exempted from the regulatory (coercion) mechanisms currently in place such as requirements for physical presence before accessing salary.

Third, at the facility level, the two above factors were influencing the attitudes of local health facilities and services managers towards absentees. Some of them paid no attention to management issues of the new HCWs either by the fear of receiving blame from supervisors (at the district, regional or central level) or for not favouring double standards in the management of the personnel.

*... We have visitors here not health workers... They come to visit us at the end of the month to benefit their salaries and go back to Conakry... We can’t blame them because they make the effort to come at the end of the month unlike others who left months ago and in full view of all... (IDI# 65 local health facility manager)*

### Intention to quit in the next 12 months

**Fig. 7:**
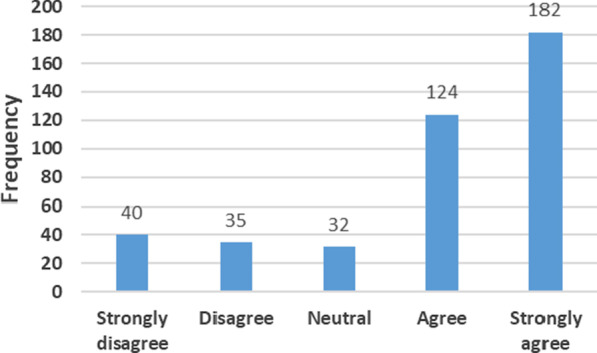
Frequency distribution of responses to the question: “Do you intend to remain in your present position for the next 12 months?” *N* = 413

Overall, only 18% of HCWs present at assigned post 12 months after deployment expressed their intention to quit their position in the next 12 months (Fig. 7). The private health sector and government health facilities in other locations of the country were commonly cited as their potential destination.

We identified six main factors underlying the intention to stay for the next 12 months. Some of the factors are inter-related: the sense of engagement with the state, the fear of losing the job, deployment to a preferential zone, support and cooperation of the spouse, and the holding of a position of responsibility.

For instance, some participants reported the fear of losing their job as a reason for staying at their current position. For participants of this group, they would have been more motivated to work if they were deployed to their preferential district.

*I was indeed ready to come to the rural zone but I would prefer to be deployed to Kissidougou [another rural district, 601 km far from Conakry, the capital] where my mother stays, or to Siguiri where two of my brothers live... But I had to come if not I would have lost my job... (IDI#52 health provider)*

Similarly, the local salary payment serves as a coercive measure constraining health personnel to work in the deployed zones.

*... Now we have a means of pressure on them [health workers] which is the salary... Several of them [health workers] had left their posts for unknown reasons and since they were prevented from receiving their salary, they have returned... At the moment, there is even a woman who had joined her husband and stayed there for more than 4 months; her salary is being frozen. Once back, she will have to stay at her post for at least 60 days before she has access to her entire salary... (IDI#89 local health authorities)*

Also, the agreement and support of the family, especially the spouse, was reportedly an influencing factor for taking up services. Many of these participants were married women and stated that the encouragement and support of their spouses in “family re-organization” was crucial in this process.

*My husband was cooperative... He supported me and encouraged me to take up my functions here... The only challenge we faced was how to reorganise our family to the actual situation... We agreed that I came with our last born and took the two others to my sister... She is a teacher and would take care of them as her own... (IDI#21 health provider)*

## Discussion

In this section, a three-way discussion of the findings is undertaken, following the study objectives. First, the effects of the deployment of HCWs in terms of need-based distribution and relevance to the needs of the local health systems; second, the analysis of the post-Ebola retention policy; and finally, absenteeism of HCWs one year after their deployment.

### Effects of the deployment of HCWs

Our finding on the distribution of HCWs shows that some of the initial objectives of the post-Ebola HCWs reform were achieved. First, more than two-thirds of the workforce were assigned to primary healthcare delivery facilities, predominantly located in rural and underserved settings where the majority of the Guinean population stays and where healthcare delivery capacities were previously substantially limited because of the shortage of HCWs [4, 21]. A plausible explanation of this finding could be the decentralization of the HCWs’ posting functions to local district health authorities. Indeed, before the Ebola outbreak, HCWs were directly posted by the central administration of the MoH without prior consultation with, and need-based assessment of the local health system authorities [22]. In 2011 for instance, 53% of the 1,240 HCWs recruited were directly posted, by the MoH, to health facilities located in urban areas; contributing to the accentuation of the geographical maldistribution of HCWs between rural and urban areas. Other studies have described the impact of decentralization of HCWs functions, including the posting, on ensuring a responsive distribution of HCWs [23, 24].

Second, the data show that primary health facilities were mostly staffed by formal skilled HCWs. A valuable component of this was the medicalization of almost nine out of ten health centres in the study sites. Evidence sustains that the medicalization of health centres expands the range of health problems to be addressed at primary levels, increases the quality of service provision, and restores credibility to the health system [25]. Further investigation is needed to explore whether the “quality improvement” in staffing levels of primary health facilities reported in this study would lead to improved access and quality of care in rural Guinea in the context of lack of financial protection in access and utilization of healthcare services.

However, results revealed several challenges local health authorities and services managers faced to reorganize the health services and retain some HCWs in public care delivery following the posting of the new workforce. One of the challenges found and emphasized by local stakeholders was the gender imbalance with many deployed female HCWs, alongside their perceived challenges to cope with living and working conditions in rural areas. Previous studies in Niger, Ethiopia, and Rwanda have shown that female HCWs are less inclined to work in rural areas compared with their male counterparts [26, 27]. Local health authorities readapted to this situation by (re-) allocating many female HCWs, especially pregnant and breastfeeding women, to urban and easy-to-access rural areas. This was likely done to facilitate their mobility as cultural norms pertaining to most West African countries support that married female HCWs are expected to follow their husbands [27]. Nonetheless, in the Guinean context, and as shown in this study, female HCWs exclusively make up 100% of midwives and an overwhelming majority of nurses specialities (assistant-nurses and nurses); however, they are the main providers of maternal and child care [3]. This finding puts a spotlight on the need for rethinking the recruitment and deployment strategies of the workforce in Guinea to optimize their effects and (future) impact on the local health system and population health. This is all the more necessary as the medical sector is increasingly becoming, economically, less attractive to the male gender [28, 29]. Creating a favourable environment for female HCWs, through the testing of strategies, such as the rural pipeline suggested by the WHO—which consists of training HCWs locally, recruiting, maintaining, and providing them with the necessary support needed for the fulfilment of their roles but within their usual environment—would increase their ability and motivation to work in rural settings [27, 30–32]. In Burkina Faso, for example, the regionalisation of the recruitment of socio-professional categories dominated by females HCWs like nurses, midwives, and auxiliary midwives was proven effective in retaining HCWs in rural areas and correcting the uneven distribution of HCWs in some regions [33]. Besides, evidence sustains that if placed in favourable conditions, female HCWs are likely to provide quality care, comply with guidelines, write fewer prescriptions, and refer cases more often compared with their male peers [34].

Local stakeholders faced difficulties in managing the former HCWs, especially volunteers and contractual workers, to make them adhere to the posting of the new workforce. Reasons for this included their frustration for not being recruited and the loss of positions of responsibility, resulting in job abandonment in the public sector and collaborative conflicts among HCWs. This is of particular concern given the role of volunteers and contractual workers in the Guinean health system [4]. Several strategies were adopted by local health authorities to mitigate these unintended effects of the posting of HCWs including the transfer of volunteers and contractual workers to peripheral health facilities and incentivising them with financial bonuses generated through their activities. In the context of lack of social security, poor supervision, and management of HCWs in Guinea, such strategy may expose the rural population to higher informal out-of-pocket payment incompatible with their economic status and the initial objectives of the post-Ebola health system reform. [4, 21, 35]

### Analysis of the post-Ebola retention policy

We analysed that HCWs are strongly motivated to practice in rural settings during their recruitment; however, this motivation is reversed along with their deployment, installation, and integration phases. Reasons for this include the mismatch between personal choices and actual places of deployment, professional dissatisfaction, and inadequate living conditions.

Findings show that HCWs’ attraction in rural Guinea is strongly affected by the desire to become a public servant and the learning possibilities that rural areas offer. These findings concur with other studies conducted in rural Senegal and Niger [36, 37]. This motivation, in our context, stems from labour market features, which are characterized by a chronic oversupply and underemployment of HCWs alongside difficult learning possibilities in urban health facilities—as a consequence of high staffing levels of healthcare facilities—especially in the capital Conakry.

Shortly after their recruitment, participants indicated that factors such as the deployment in non-preferential locations, and for married women, challenges for family reorganization, and the opposition of spouse contributed to their discomfort towards joining their assigned locations. Nevertheless, this seems not to affect services take-up which was as high as 98%.

A total of 18% HCWs intended to quit their post in the following 12 months. Private sector or public facilities in other locations different from the present were reported as potential destinations of the HCWs. This finding concurs with that of Reid et al. in 2018, which reported that many health care workers intended to migrate or go into private practice after their assignment to rural settings [38]. This finding is however different from that of Frehywot et al. who reported in their review an increased willingness of HCWs under retention programmes in Indonesia and Thailand to stay in their assigned post, even beyond the recommended period [39]. In Zambia meanwhile, Gow et al. reported in their study that many HCWs expressed their desire to quit government services after their deployment [40].

Overall, barriers for HCWs installation and integration in the current study seem to be multifaceted and related to the structural (housing, electricity, potable water, and schools for children), organizational (salary levels and payment methods, difficult learning environment, and inadequate supportive management), socio-cultural (poor health-seeking behaviour leading to under-utilisation of health services and demotivation to stay in assigned locations) environments in which the local health operate as well as personal characteristics of the workforce (gender, living standards, and rural background). In response to these barriers, various coping strategies and mechanisms were undertaken by the workforce (seeking advice and focusing on healthcare provision in order to avoid conflict of collaboration with former HCWs), local health authorities (internal rotation, reorganization of services taking into account social endeavours of HCWs), and communities (provision of food and housing) to support the staff and facilitate its installation and integration. Despite the positives discussed above, this post-Ebola HCWs retention programme tends to privilege the “one size fits all” approach in the installation and integration phases of HCWs. This approach consists of considering salary only sufficient for motivating and retaining the workforce in rural areas. Indeed, the on-going retention programme in Guinea excluded financial (hardship allowances) and non-financial (training and career development prospects) incentives allocation as core components of the successes of several rural retention policies in Western Africa and beyond [30]. For instance, financial compensation for geographical distances, living and working conditions for health professional working in rural Niger improved their availability and retention between 2005 and 2008 [41]. In Mali, meanwhile, the programme for the medicalization of rural health areas retained, for four years, medical doctors because of its training package [25, 42]. The provision of housing and children scholarships encouraged health professionals to stay in rural Kenya and Zambia [39].

### Absenteeism of health workers

Evidence from previous studies in Guinea have shown an absenteeism rate of ~ 43% among public HCWs, and the underlying factors for this included low salary—pushing them to a dual practice phenomenon only possible in urban areas, and the centralized management of HCWs including salary payment [4, 21]. However, despite the delocalization of salary payment and the 40% increase in the salary level of the workforce in the post-Ebola context, national supervision conducted in November 2017 (seven months after deployment) by the MoH, in our study sites, showed a 20% absenteeism rate among HCWs [43]. This is relatively lower compared with the 31% absence rate observed in our study. The unofficial and unannounced characteristics of our data collection, as opposed to national supervision, could explain this difference in absenteeism rates. Other authors have previously reported higher absence rates in five developing countries including Uganda, Bangladesh, and Peru during unannounced visits compared with announced ones [44].

Our finding, however, revealed the poor monitoring and accountability of HCWs which might have led them to adopt a leave but stay tuned strategy. This strategy consists of leaving one's post without a valuable reason and joining as soon as a national mission is announced, especially in the districts surrounding the Capital. Indeed, our finding showed that up to 51% of absenteeism observed was of non-justifiable reasons; meanwhile, such reasons for absenteeism were not reported during the national supervision.

Underlying causes for absenteeism pointed out by participants included the lack of transparency and effective coordination in decision-making processes for leaves allocation. HCWs absenteeism is known to compromise health system effectiveness and (quality) healthcare services delivery, and led to important economic losses, particularly in under-resourced health systems, where salaries are a substantial part of the health budget—in Guinea, 80% of the budget allocated to health goes to HCWs salaries [5, 45]. To mitigate these, the implementation of regulatory mechanisms had been proven effective in many countries. These measures vary from the “carrots” (e.g.: providing learning opportunities or financial rewards for good attendance) to the “sticks” (e.g.: attendance policies, documenting the process for absence review, defining disciplinary procedures for absence, monitoring, audit, and dismissal or forced retirement) methods [20, 45–47]. In the weeks following the national supervision of November 2017, 92 HCWs were dismissed nationwide from the public services as a result of, among others, their absence at their assigned posts (42%) or being non-healthcare professionals (32%). These 92 people were replaced, in the public health workforce payroll, by other HCWs. However, until January 2021, we had not been aware of any other national supervision undertaken by the MoH. This infrequent national supervision of HCWs—plausibility due to financial constraints—raises the need for empowering local authorities, including actors outside the health sector such as community representatives, in the management of the HCWs in Guinea.

Furthermore, it appeared that no policy and guidelines existed to orientate the post-Ebola retention policy in Guinea. Although this was not the focus of our study, it emerged as an important aspect during the research. The lack of policy and guidelines on the on-going programme has led to some confusion of roles and responsibilities between central and local stakeholders in the decision-making processes regarding the management of HCWs. For example, there existed no clear principle guiding and documenting the redeployment and allocation of training leaves by central administration. This may undermine the earlier positive effects generated by this policy. However, a national policy of HCWs development was elaborated in July 2019 with the aim of ensuring, by 2024, the availability—in adequate quantity and quality, in all professional and technical positions—of well-motivated HCWs who are individually and collectively committed to performing their duties in a decent work environment [48]. A further step to this policy development would be the elaboration of a strategic plan for the development of HCWs in the post-Ebola context including a comprehensive detail of the on-going programme for meeting the needs of HCWs in rural Guinea. A clear distinction of roles and responsibilities of central and local actors (in and outside the health sector) needs to be ensured to avoid incoherent decision-making in the management of HCWs (e.g.: leaves allocation). Moreover, the development of guidelines on primary healthcare (immunization, maternal and child health, nutrition, healthy lifestyle, etc.) is needed to support the management capacities of local health services managers. Finally, the development of job descriptions of HCWs needs to be done in order to alleviate or minimize the underlying factors and effects of collaboration conflicts between professionals.

### Study limitations

This study has some limitations. First, the analytical framework used to guide this research is on the retention of HCWs, and not necessarily for evaluation of the implementation process of HCWs retention policy. Additionally, no baseline data were collected at the beginning of the deployment to include all HCWs who took up duties. If done, this would have allowed a comparison between baseline data and data collected one year after. This fact might have led to the underestimation of certain findings such as the intention to quit or dissatisfaction with the professional situation. Explicatively, it is likely that HCWs present on the study sites during data collection were more satisfied with their living and professional situation and therefore, less inclined to quit their job compared with those absent. Second, the subjective reporting of reasons for absenteeism could constitute an information bias. Indeed, in the absence of administrative documents justifying the absenteeism of HCWs, health facilities, and services managers were the main sources of information for this study variable. We also failed to contact the new HCWs absent from their post during data collection. If done, this would have helped to cross-check the reasons for absenteeism reported by health services and facilities managers. Third, this study took place in a moment where HCWs turnover was a sensitive issue for both local and national actors. It is, therefore, possible that some HCWs were contacted by their managers or colleagues to temporarily re-join their positions during the data collection period. This may have led to an underestimation of the absenteeism rate reported in this study. Another limitation, not the least, is inter-coding biases which should have been addressed by having two or more people coding the interviews separately and agreeing on findings upon team consultation. Because of time constraints, only one interviewer could code the interviews. Finally, as interviews were translated from French to English, possible bias related to language interpretation might be associated with findings.

This study’s strength resides in its mixed-method design (qualitative and quantitative) and the diversity of the data collection tools and study participants that allowed triangulation [49]. The data were collected by a research team familiar with qualitative research methods and the local context. Besides, this research covered five rural health districts with different socio-demographic, geographical, and economic characteristics. Therefore, the transferability of findings to nationwide relevance for Guinea should be possible.

## Conclusion

This study is the first to document the implementation process of the post-Ebola healthcare workers retention policy in rural Guinea. This programme appears to have been successfully positive and met some of its initial goals of the redistribution of HCWs and the quality improvement of staffing levels (e.g. the medicalization of health centres) in peripheral healthcare facilities, and the enhancing of district health offices’ capacities (e.g. timely reporting of data). The delocalization of salary payment has relatively empowered local health authorities in the management of healthcare workers, and salaries serve as an instrument for the regulation of health workers’ absenteeism. As the results show, however, the effects of this programme could be improved by prioritizing local recruitment and deployment of healthcare workers and developing strategies to mitigate HCWs absenteeism. More attention should be given to the development of policy and guiding documents with the full participation of all actors (including local stakeholders) and a clear share of their roles and responsibilities for improved implementation and efficacy of this programme.

## Supplementary Information

**Additional file 1: Annex S1.** Data collection tools.

## Data Availability

The datasets used during the current study are available from the corresponding author on reasonable request.

## Footnotes

1 Healthcare workers holding the status of civil servants by virtue of which they have the notoriety—compared to volunteer and contractual workers—to practice fully in the public health facilities where they are assigned.
