# Supplementary material for: Retention of healthcare workers 1 year after recruitment and deployment in rural settings: an experience post-Ebola in five health districts in Guinea
Source: Hum Resour Health. 2021 May 17;19:67. doi: 10.1186/s12960-021-00596-x (PMC8127209; doi:10.1186/s12960-021-00596-x)
Supplement: Supplementary file 1 — Additional file 1: Annex S1. Data collection tools. [file 12960_2021_596_MOESM1_ESM.docx]

**Annex 1 : data collection tools**

## Interview guide

1. Responsible at all levels :

- Have the lessons learned from the evaluation of previous strategies been taken into account in this new HRH recruitment strategy?
- Why was it necessary to implement this new strategy?
- Through which institutional arrangements has this policy been implemented?
- What is the governance/management/coordination mechanism put in place to support this strategy? (Are there management committees at local or regional level? Who are the players involved in this support? What are their roles? Does the local level have decision-making power in the implementation of this strategy? What is its role?)
- What are the means or sources of funding to support this strategy? (Is there sufficient budgetary space? Partners' contribution? Support mechanism?
- What are your experiences in terms of successful HRH (re)distribution and retention strategies in Guinea or elsewhere?
- What was the context, the actors, the monitoring mechanism of this strategy for its success?
- What are the constraints/enablers observed in the implementation of these strategies in terms of (re)distribution and retention of HRH?
- What are the personal, socio-cultural, economic, geographic, and professional factors influencing this (re)distribution/localization of HRH at the local level?
- What is your opinion on the current management of the salaries of new health personnel?
- What do you propose to improve this?
- What remedial action can be undertaken to address each of the above difficulties?
- Regarding the reassignment of new health personnel, where do you think the problem exists ("central", "intermediate", "local" including communities)?

2. Health care providers

1. General question :

- Through which institutional arrangements has this policy been implemented?
- How is the service administratively organized and provides health services related to this recent deployment? How are tasks reorganized as a result of this deployment? How collaboration with other colleagues is organized (conflicts of skills, conflicts of interest, between old and new staff and the public, private and NGO sectors in terms of human resources including remuneration, health service delivery)?
- Does this suit you or do you have proposals to improve this?
- What is your assessment of the work atmosphere? The role of the other health workers in the team in terms of task sharing, knowledge and experience, solidarity, support? The role of managers in the management of health workers, taking into account concerns about career planning, continuing education, creating a pleasant working and living environment?

II. Career path of the training:

- What is the last level of education you completed prior to your health training and where did you reside prior to this training?
- What motivated your choice of health training?
- In this type of structure did you do your health training (public/private)?
- What is your region of habitual residence?
- How and where were your training courses organized?
- In an urban area exclusively? In a rural area exclusively? In its area of residence exclusively? Outside your area of residence?
- Has your training prepared you to work at your current post?
- If yes, how did it prepare you and how? (Community commitment approach, health promotion?)
- If not, what can be done to improve this?
- Did your basic training (theoretical/practical) meet your expectations? What were your expectations?

III. Professional background:

1. What happened after the training?

- When did you start your first internship and/or job? How do you feel about the time between the end of your health training and your start date?
- In which sector (private "clinical, project/public") were you working? For how many years?
- Did the job or internship meet your expectations in terms of acquired skills, experience and remuneration?

1. Have you or any of your colleagues worked in an Ebola control programme? Could you explain the difference in treatment between this programme and the State? Could this have an impact on the retention of health workers?

- IV. Recruitment announcement and transfer:
- How have you been informed of the public service recruitment competition? How did your recruitment go (constitution of your files, conduct of the test)?
- Where were you living at that time?
- What were your motivations for taking this test?
- What do you think of the organisation of the competition (from the call to the result in terms of transparency, facilitating the process, level of information, support from local authorities)?
- What was your perception of the five-year commitment to work within the country?
- What is your assessment of the waiting time for the results of the competition?
- While you were waiting, what were you doing? Did you have a job? Where did you live?
- How did you welcome the announcement of the results?
- What do your colleagues/colleagues who were working as trainees’/contract workers in health services and were not recruited think? What impact did this have on their motivation to work in the health services?
- How did you welcome the decision to transfer? Did your transfer area meet your expectations? Why/why not? What happened afterwards?
- How was your taking up organized/planned?
- Did you benefit from the support measures to facilitate your taking up this position?
- If yes, what kind of measures?
- If not, has it had an impact on your taking up? Is it related to a health problem? Which one? How do you manage this illness?
- Are you married or in couple?
- If yes, since when?
- What positive or negative role does your spouse play in your work?
- Had your spouse helped/encouraged you to apply for this recruitment test, to take the job, to stay at your job?
- Does your spouse help you with childcare, lightening your family duties?
- Do you have any suggestions for improving the recruitment and deployment of health workers within the country?

V. Reception and Installation:

1. How do you rate your job in terms of hospitality of the communities, local authorities and fellow health care providers?

- Check whether he or she has not taken up his or her post or no longer resides in the area to which he or she was originally transferred and ask why (state of health, marital/family situation, difficulties in adapting to local living conditions or difficulties in receiving a salary)?
- Has he been reassigned elsewhere? If so, who facilitated this reassignment and for what reason?
- Does he/she receive his/her salary continuously? By what means (bank transfer, direct payment)?

*Explore further to find out if he or she is under pressure to return to work.*

1. What are the enablers or difficulties encountered during your integration?

- How do you adapt to local cultural, socio-economic and professional realities?
- Culturally, explore the role of religion, food, clothing, customs and mores on its integration;
- Socio-economically, explore whether he speaks the local language, whether he makes new friends easily, whether his salary covers his needs (food, housing, health, education of children...).
- Professionally, explore whether his work environment (collaboration with managers and former staff) is conducive to his integration.
- Do you have any side activities to help you get by?

1. What are your expectations/prospects (e.g. in terms of continuing education, promotion, etc.)? How long do you plan to stay here?

- What are the factors that motivate you to stay in your posting area?
- What are the factors that demotivate you to stay in your place of posting?

1. What should be done to keep you in your posting area as long as possible?

- Do you have any other ideas on how to improve the retention of health workers in rural areas?

1. What is your perception of the method of payment of salaries currently in force in the context of this new recruitment and deployment strategy? Do you have any other proposals to improve this?
2. Are you aware of the reassignments of new health staff? What is your assessment of these reassignments? At what level is the problem located (central, intermediate, local level including communities)?
3. Community

Recruitment and posting:

- What do you think of the organisation of the competition (in terms of transparency, response to community demand)?
- What is your perception of the five-year commitment of medicine in the country?
- Have you played a role in the recruitment of health workers to the civil service? (Trainee/contractual health workers working in your community?)
- How did you welcome the announcement of the results? (Transparency, priority of communities taken into account?)
- How did you welcome the decision to transfer new health workers to your community? What happened afterwards?
- After you were transferred, what happened? How did you organize/plan the takeover of these new health workers?
- Did you benefit from the accompanying measures to facilitate this new function?
- If yes, what kind of measures?
- If not, did it have an impact on their taking up their posts?

Reception and Installation:

- How was the reception and installation of the new health workers? What was the role of the community and community leaders? Local authorities? Other actors were involved; who and how?
- What are the enablers, constraints and difficulties encountered during your integration/installation?
- How do you adapt to local realities?
- What are your expectations/perspectives? How long do you plan to stay here?
- What are the factors that promote the retention of health workers in your locality/community?
- What are the factors that hinder the retention of health workers in your locality/community?
- What should be done to improve health worker retention in your locality/community?
- Do you have any other ideas on how to improve health worker retention in your locality/community?

Are you aware of any reassignments of new health care personnel? What is your assessment of these reassignments? At what level is the problem located (central, intermediate, local level including communities)?

## Questionnaire

Socio-demographic characteristics of health personnel

Date of birth___/____/______

Gender: Male Female

Professional category: *(doctor, nurse, midwife, ATS, laboratory technician, public health technician, etc.)* _____________________________________

Place of residence before recruitment: _________________________________

Posting place *(district sanitaire)* ___________________________

Did it meet your expectations? Yes No

*6.a. If not, what was your preferential posting place? _____________*__

Posting area *(urban or rural municipality)* ___________________

Marital status prior to recruitment (married, single)*, etc._____________*_____

*8.a. If married, what is the spouse's place of residence? _________________*

*8.b.* *If married, what is the spouse's profession? ________________*

Number of persons in charge (children, parents, friends...) ____________

10. Current marital status (married, single, etc.) *___________*_________________

Do you reside with your spouse? Yes No

II. School curricula for health personnel

12. Date of completion of studies (day/month/year) _____/_______/_________

Type of health school/university attended: Private Public

In which city did you reside during your medical training or health school? ______________________

In which city did you reside during your high school (college/high school) education? ________________________________

In which city were your internships organized frequently? ______________________

In which health structure were your internships organized frequently? (University hospital, CHC, HC, Hospital, NGO, Project, International Institution) _______________________________

In which health district/commune did you carry out your clinical synthesis/end-of-study internship? _____________________________

What is your highest diploma/certificate? *(Doctorate, State Nurse, ATS, Certificate of Completion of Medical Studies, Bachelor's Degree...)* ___________________________________

What is your current rank in the Civil Servant (A, B, C, D)? _________________________

Other academic training (University degree, bachelor's degree, master's degree, doctorate) *[specify the title of the highest degree even if it is in a field other than health].* __________________________________________________

Describe your level of Computer literacy.: (put a cross through)

| Fields | Level of software expertise | | | | |
| --- | --- | --- | --- | --- | --- |
|  | None | Beginner | Middle | Good | Excellent |
| Use of the Internet |  |  |  |  |  |
| WORD Software |  |  |  |  |  |
| EXCEL Software |  |  |  |  |  |
| POWER-POINT software |  |  |  |  |  |
| ACCESS Software |  |  |  |  |  |
| EPI-INFO |  |  |  |  |  |
| R (or) Stata (or) SPSS (circle) |  |  |  |  |  |

III. Knowledge of the Civil Servant Recruitment Process

How did you obtain information about this recruitment to the Civil Servant (friend, relative, radio, telephone operator, television, email, poster, etc.)? ___________________________________

Where were you residing at that time? ________________________________

How were you recruited into the Civil Servant? (Test, on Ebola basis) _______________________________

*25.a. If recruitment by test, what was your test centre? _______________*

*25.b. If recruited on behalf of Ebola, what structures/institutions did you work with in the response to Ebola disease? __________________________*

Information on the process of deploying new personnel

How were you informed of your posting area (friend, relative, radio, telephone operator, television, email, poster...)? ____________________________

Were you informed that after recruitment you would be posted to a rural area?

Yes No

Did you know that new personnel would stay 5 years in their assigned post before applying for a new transfer?

Yes No

*28.a. If yes, did you sign an agreement to this effect?* Yes No

Are you currently in the Health district of your first posting? Yes No

*29.a. If not, what was the health district of your first posting? _______*

What is the date of your posting to your current post? ____/____/_____

Date of service taking up in your current health district? _____/______/______

What position do you currently hold? __________________________________

V. Reception and Integration of New Health Personnel

33. Clearly answer the following questions using the following scale:

*1= Strongly Disagree; 2= Disagree; 3= Neutral; 4= Agree; 5= Strongly Agree*

| AFFIRMATIONS | | Your response |
| --- | --- | --- |
| When you came to take up service, you were well received by the health authorities (DPS, etc.) and they continue to support you. | |  |
| When you came to take up service, you were well received by the communities and you think this continues to be the case | |  |
| You have been well received by the former staff you have found on the site and the collaboration is good. | |  |
| You are satisfied with your current living conditions (housing, food, water, transport, electricity, telephone, internet) | |  |
| Since taking office, you have had no difficulty receiving your salary on time. | |  |
| Since taking up service, you have been satisfied with your current professional situation. | |  |
| There is an atmosphere of peace, stability and security in your work place. | |  |
| Overall, your welcome and integration have been good here. | |  |
| You intend to remain in your present post for the next 12 months. | |  |
| What are the three factors, in order of importance (1=most important) that will motivate you to stay working in the rural areas? | 1_________________________  2_________________________  3_________________________ | |

VI. Work experience before recruitment

Before this recruitment, did you work in the health sector? Yes No

*34.a. If not, what were you doing? __________________*

*34.b. If yes, what work were you doing? _______________________________*

*34.c. Where did you work? __________________________________________*

*34.d. Specify the area (rural, urban)? _______________________________*

In which sector did you work (private/public/mixed)? ______________________

*35.a. What type of structure did you work in? (University hospital, Hospital, CHC, HC, NGO, Project, International Institution)* _______________________________________________

*35.b. How long did you work at this job? ________________*

Overall, how many years of experience did you have before you were recruited? ________

In addition to your current job, do you currently do any private work? Yes No

In addition to your current job, do you currently do any work in the private sector?

Yes No

ANC and Childbirth: Yes No Primary Curative Consultation (PCC): Yes No

Specialist Consultation: Yes No Routine immunization: Yes No

Coordination/supervision: Yes No Follow-up/Evaluation/Research: Yes No

HIV management : Yes No Diabetes Management: Yes No

Other, please specify _____________________________

Since taking up the job, have you received any training? Yes No

*39.a. If yes, in which fields* _____________________________________________

________________________________________________________________________

If you receive a job announcement from an international NGO, will you give up your current job?

Yes No Maybe

And if you were offered an opportunity to work outside the country now, would you give up your current job?

Yes No Maybe

Some of the new officials did not stay in their posts within the country or did not take up their posts. In your opinion, what are the main reasons for this?

*1__________________________________________________________________________*

*2__________________________________________________________________________*

*3__________________________________________________________________________*

*4__________________________________________________________________________*
